# Pharmacist gender and physician acceptance of antibiotic stewardship recommendations: An analysis of the reducing overuse of antibiotics at discharge home intervention

**DOI:** 10.1017/ice.2022.136

**Published:** 2022-06-07

**Authors:** Valerie M. Vaughn, Daniel L. Giesler, Daraoun Mashrah, Adamo Brancaccio, Katie Sandison, Emily S. Spivak, Julia E. Szymczak, Chaorong Wu, Jennifer K. Horowitz, Linda Bashaw, Adam L. Hersh

**Affiliations:** 1 Division of General Internal Medicine, Department of Internal Medicine, University of Utah School of Medicine, Salt Lake City, Utah,; 2 Division of Health System Innovation & Research, Department of Population Health Science, University of Utah School of Medicine, Salt Lake City, Utah,; 3 Division of Hospital Medicine, Department of Internal Medicine, Michigan Medicine, Ann Arbor, Michigan,; 4 Department of Pharmaceutical Services, Michigan Medicine, Ann Arbor, Michigan,; 5 Division of Infectious Diseases, Department of Internal Medicine, University of Utah School of Medicine, Salt Lake City, Utah,; 6 Department of Biostatistics, Epidemiology and Informatics, University of Pennsylvania Perelman School of Medicine, Philadelphia, Pennsylvania,; 7 Division of Epidemiology University of Utah, Salt Lake City, Utah,; 8 Clinical Experience and Quality Program, Department of Internal Medicine, Michigan Medicine, Ann Arbor, Michigan; 9 Division of Infectious Diseases, Department of Pediatrics, University of Utah School of Medicine, Salt Lake City, Utah

## Abstract

**Objective:**

To assess association of pharmacist gender with acceptance of antibiotic stewardship recommendations.

**Design:**

A retrospective evaluation of the Reducing Overuse of Antibiotics at Discharge (ROAD) Home intervention.

**Setting:**

The study was conducted from May to October 2019 in a single academic medical center.

**Participants:**

The study included patients receiving antibiotics on a hospitalist service who were nearing discharge.

**Methods:**

During the intervention, clinical pharmacists (none who had specialist postgraduate infectious disease residency training) reviewed patients on antibiotics and led an antibiotic timeout (ie, structured conversation) prior to discharge to improve discharge antibiotic prescribing. We assessed the association of pharmacist gender with acceptance of timeout recommendations by hospitalists using logistic regression controlling for patient characteristics.

**Results:**

Over 6 months, pharmacists conducted 295 timeouts: 158 timeouts (53.6%) were conducted by 12 women, 137 (46.4%) were conducted by 8 men. Pharmacists recommended an antibiotic change in 82 timeouts (27.8%), of which 51 (62.2%) were accepted. Compared to male pharmacists, female pharmacists were less likely to recommend a discharge antibiotic change: 30 (19.0%) of 158 versus 52 (38.0%) of 137 (P < .001). Female pharmacists were also less likely to have a recommendation accepted: 10 (33.3%) of 30 versus 41 (8.8%) of 52 (P < .001). Thus, timeouts conducted by female versus male pharmacists were less likely to result in an antibiotic change: 10 (6.3%) of 158 versus 41 (29.9%) of 137 (P < .001). After adjustments, pharmacist gender remained significantly associated with whether recommended changes were accepted (adjusted odds ratio [aOR], 0.10; 95%confidence interval [CI], 0.03–0.36 for female versus male pharmacists).

**Conclusions:**

Antibiotic stewardship recommendations made by female clinical pharmacists were less likely to be accepted by hospitalists. Gender bias may play a role in the acceptance of clinical pharmacist recommendations, which could affect patient care and outcomes.

Clinical pharmacists play a vital role in the oversight and delivery of antibiotic stewardship interventions in hospitals, particularly in small or rural hospitals where infectious diseases specialists are limited.^1^ The most effective antibiotic stewardship interventions, such as postprescription audit and feedback, involve stewardship personnel recommending to prescribers that they modify a decision about an antibiotic. These recommendations are often unsolicited, made by someone external to the clinical team, and they are variably accepted by prescribers.^2–5^ Although methods exist to improve the delivery of recommendations by stewards to prescribers, such as “handshake stewardship,”^6^ the way the advice is received by the prescriber is crucial to stewardship effectiveness.^7^ Although a steward of any background can be seen as an outsider to the clinical team,^8^ additional power differentials can lead to conflict between pharmacists and physicians in the context of stewardship recommendations.^7^

Trust and willingness to accept recommendations can differ based on demographic characteristics of the person making the suggestion, including gender.^9,10^ Bias, implicit or explicit, can influence prescriber acceptance of steward recommendations. Gender stereotypes, or beliefs about a person’s capabilities or attributes based on their apparent gender, can have detrimental effects on collaborative work,^11^ including in medicine.^12^ For example, a prescriber may discount the expertise of a female steward or find her presentation of information less credible relative to an equal level of expertise or information communicated by a male steward. To determine whether steward gender is associated with prescriber acceptance of stewardship recommendations, we compared the effectiveness of a discharge antibiotic stewardship intervention by pharmacist gender.

## Methods

To evaluate the association of pharmacist gender on acceptance of antibiotic stewardship recommendations, we retrospectively evaluated the effectiveness of the (Reducing Overuse of Antibiotics at Discharge (ROAD) Home intervention.^13^ The ROAD Home intervention occurred between May and October 2019, in which clinical pharmacists led an intervention to improve antibiotic prescribing at discharge at a large, US academic medical center. Prior to the intervention, clinical pharmacists were commonly involved in inpatient antibiotic stewardship efforts (eg, monitoring vancomycin levels, and/or performing postprescription audit and feedback of selected antimicrobials), but they did not have a standard process for discussing or providing recommendations for antibiotics at discharge. Although all clinical pharmacists had explicit and implicit roles in antibiotic stewardship as part of their job description, they were not required to have specific training in antibiotic stewardship. Specifically, none of the clinical pharmacists were ID pharmacists (ie, had specialist postgraduate infectious disease residency training). At baseline, the study hospital was classified as “high performing” for appropriate prescribing of antibiotics, and this facility had a long history of collaborative stewardship interventions between hospitalists and pharmacists.^14–16^ Notably, strong relationships existed between hospitalist physicians and the clinical pharmacists, likely attributable to daily in-person rounds.^13^

For the ROAD Home intervention, patients were eligible for inclusion if hospitalized on a hospital medicine service (in the US hospitalists are inpatient general medicine physicians) and were receiving antibacterial therapy. All hospital medicine teams were nonresident, single-physician teams. To help provide guidance for the intervention, the antibiotic stewardship team (consisting mainly of infectious diseases pharmacists and physicians) and the study team collaborated to generate a pocket card with discharge antibiotic recommendations for the most common inpatient diseases (Supplementary Fig. 1 online). The pocket card was widely distributed to hospitalists and clinical pharmacists and was paired with education at regularly scheduled group meetings.

The 6-month intervention consisted of an antibiotic timeout (ie, structured conversation to review antibiotic appropriateness) during pharmacist rounds with the hospitalists. Rounds typically occurred in person in the hospital medicine team room and lasted ~15 minutes, during which the pharmacists and hospitalists reviewed their list of patients and discussed pertinent patient issues. The ROAD Home intervention consisted of adding antibiotic timeouts during these rounds for any patients who were anticipated to be discharged within 48 hours. The timeouts were prompted and led by clinical pharmacists who led a structured conversation with hospitalists that targeted 4 common ways to improve antibiotic use at discharge: (1) stopping unnecessary therapy (ie, antibiotics prescribed for a noninfectious or nonbacterial syndrome), (2) reducing excessive duration, (3) improving appropriate selection, and (4) documenting the antibiotic plan in the discharge summary.^14,17^

During the intervention, pharmacists prospectively recorded data on antibiotic timeouts in the pharmacist medication interventions tab in Epic software (Epic, Verona, WI) including indication for antibiotic, antibiotic prescription details (dose, frequency, and duration), whether antibiotic changes were suggested, and whether antibiotic recommendations were accepted after the timeout (confirmed by verbal agreement or change in antibiotic order). The hospitalist participating in the timeout was identified as the provider signing the progress note on the date of the timeout. Pharmacist and hospitalist gender were identified from the institution’s demographic database (self-reported). In addition, in August 2021, we surveyed all pharmacists who had participated in the ROAD Home intervention to obtain additional demographic and training information and to assess their comfort, interest, and belief in antibiotic stewardship. Surveys were conducted via Qualtrics XM (Provo, UT), with reminder e-mails to nonrespondents. For pharmacists who did not complete the survey, we searched online for data on their postgraduate training (including years of postgraduate training). Patient demographics, comorbidities, and length of stay were obtained from the institutional data warehouse. All patients with timeout data were included in analyses.

Our primary outcome was the percentage of antibiotic recommendations made by pharmacists that were accepted by hospitalists. Secondary outcomes included proportion of all timeouts that resulted in a change recommendation, proportion of all timeouts that resulted in an antibiotic change, and type of antibiotic change (discontinuation, selection, and/or duration). Descriptive characteristics were used to describe the patient cohort and primary and secondary outcomes. Bivariable analyses were conducted using the *t* test, the χ^2^ test, or the Fisher exact tests, as appropriate. We used a logistic regression model to identify the association of pharmacist gender with acceptance of antibiotic recommendations. The following patient characteristics, which could affect intervention effectiveness, were included as control variables: patient age, sex, race, Charlson comorbidity index, quick sequential organ failure assessment score [qSOFA] at 24 hours,^18^ infection diagnosis, presence of infectious diseases consultation, and length of stay. To determine whether the effect of pharmacist gender was affected by hospitalist gender, we also assessed for the statistical significance of the interaction between pharmacist and hospitalist gender. Results are reported odds ratios (ORs) or adjusted odds ratios (aORs), as appropriate.

Because our 2 clinical pharmacist project leads were male (and could have had a greater likelihood of recommendation acceptance based on their leadership role), we also conducted a sensitivity analysis excluding the pharmacist project leads. Subgroup analyses were conducted by infectious diagnosis.

We followed EQUATOR reporting guidelines (STROBE check-list in the Appendix online). Missing data were rare (2% for physician gender and “change recommended”) and were handled via pairwise deletion for bivariable analysis and listwise deletion for multivariable models. A 2-tailed α level of 0.05 was selected for all comparisons. SAS version 9.4 software (SAS Institute, Cary, NC) was used for analyses. Human-subject research approval was obtained from the University of Michigan Institutional Review Board prior to the study. Because disparities due to patient demographics may exist, we have reported data on gender, race, and ethnicity, obtained from the medical record and categorized as noted in the Appendix (online).

## Results

Pharmacists conducted 295 antibiotic timeouts during the 6-month study period. Overall, 158 (53.6%) timeouts were conducted by 12 female pharmacists and 137 (46.4%) were conducted by 8 male pharmacists. Each male pharmacist completed a median of 17 (interquartile range [IQR], 2–23) timeouts, and each female pharmacist completed a median of 4.5 (IQR, 2–22) timeouts (*P* = .67 for comparison). 4 women and 5 men responded to our survey (response rate, 45%). We found data online for an additional 4 women and 1 man. The 6 pharmacists (4 women, 2 men) with no data available were rotating pharmacists responsible for only 13 (4%) of all timeouts. Female and male pharmacists had similar years of experience and training (Table 1).

The most common diagnoses reviewed in timeouts were pneumonia (28.5%) and urinary tract infection (28.1%). Generally, patients with antibiotic timeouts conducted by male and female pharmacists had similar Charlson comorbidity index scores, qSOFA scores, diagnoses, and lengths of stay (Table 2). However, patients were younger on average in timeouts conducted by female versus male pharmacists (median age, 62.0 years vs 66.8 years; *P* = .01). Antibiotics were still prescribed at discharge after 256 timeouts (86.8%). Pharmacist gender was associated with an antibiotic being prescribed at discharge: 110 (80.3%) 137 male pharmacists versus 146 (92.4%) of 158 female pharmacists (*P* < .001). The median antibiotic discharge duration was 5 days (IQR, 3–10) with no differences by pharmacist gender (Table 2).

**Fig. 1.**
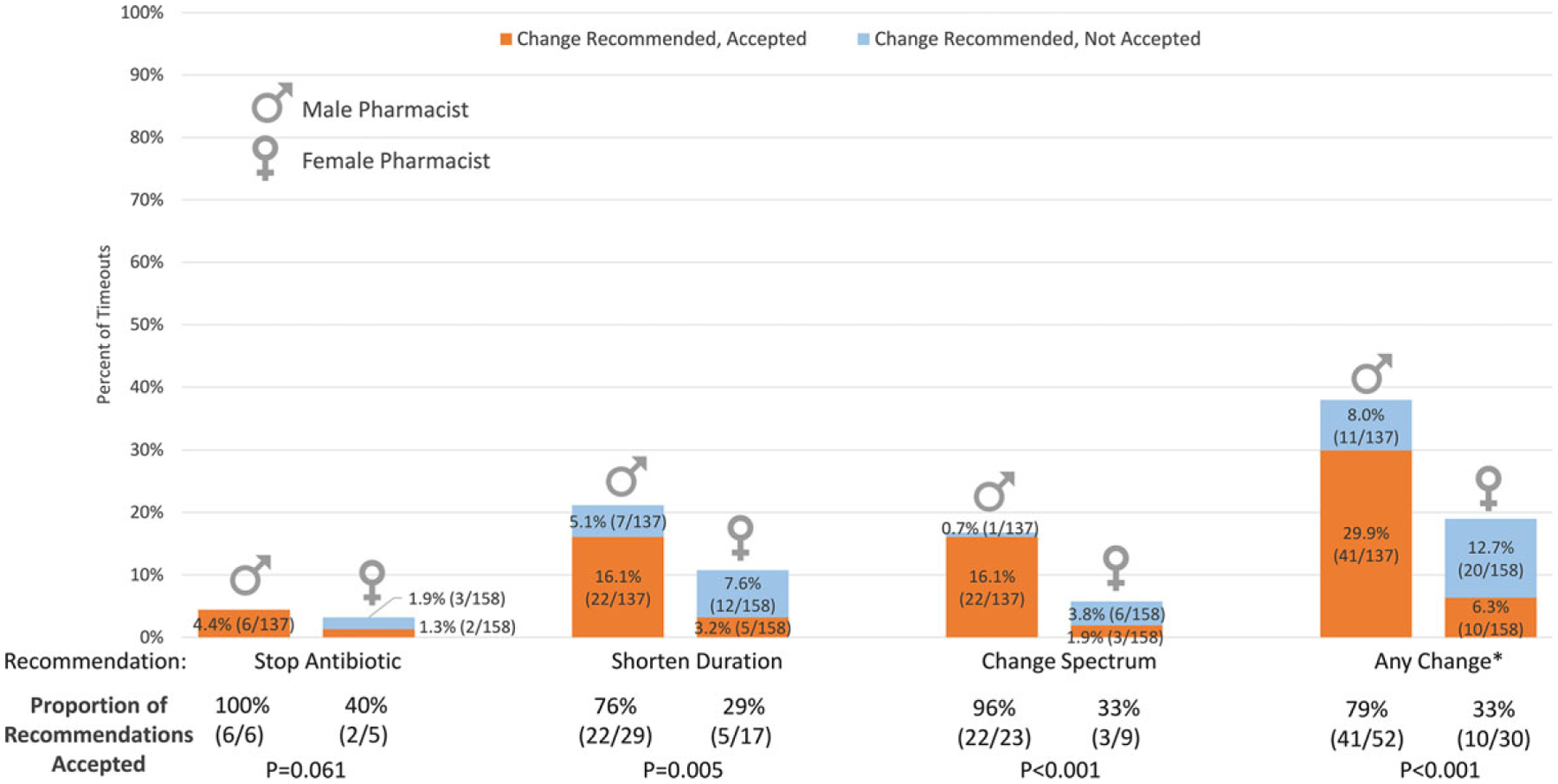
Antibiotic timeouts with change recommended and accepted, by type of recommendation (N = 295 timeouts). The percentage of antibiotic timeouts performed by male (n = 137) versus female (n = 158) pharmacists that had a change recommended but not accepted versus recommended and accepted are shown. *P* values, calculated using χ^2^ or Fisher exact test, are shown comparing proportions of recommendations accepted by gender. *Multiple changes may have been recommended or accepted during a timeout. The 6 timeouts in which 2 recommendations were made were counted as 1 total change each.

**Fig. 2.**
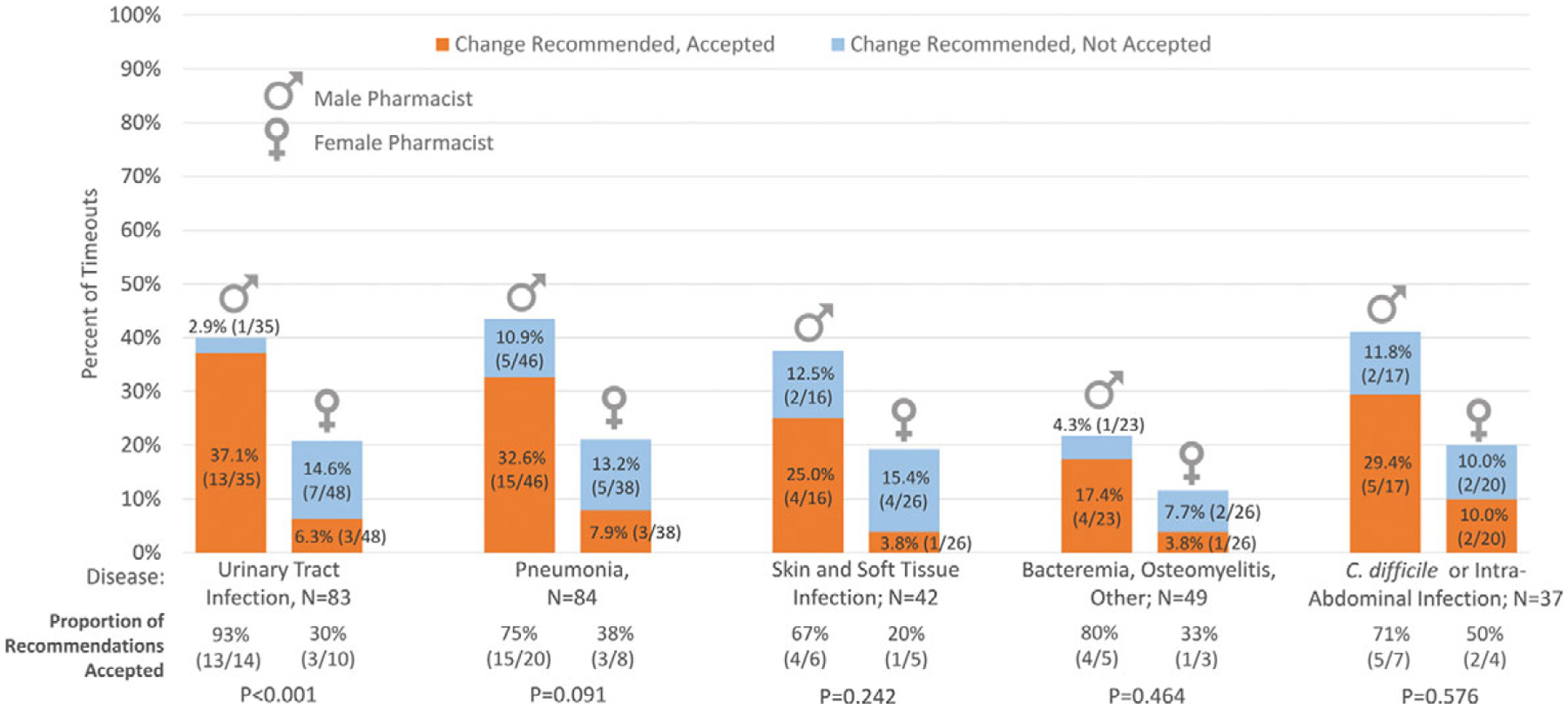
Antibiotic timeouts with change recommended and accepted, by infectious diagnosis (N = 295 timeouts). The percentage of antibiotic timeouts performed by male (n = 137) versus female (n = 158) pharmacists that had a change recommended but not accepted versus recommended and accepted are shown by infectious diagnosis. *P* values, calculated using χ^2^ or Fisher exact test, are shown comparing proportions of recommendations accepted by gender. Note. *C. difficile, Clostridioides difficile*.

Overall, pharmacists identified an issue with antibiotic appropriateness and recommended a change in 82 (27.8%) of 295 antibiotic timeouts. Of the 82 recommended changes, 51 (62.2%) were accepted by hospitalists. Thus, 51 (17.3%) of 295 total timeouts resulted in an antibiotic change. Antibiotic duration was the most commonly identified area for improvement and the most common change agreed to by clinicians (Fig. 1). On unadjusted analyses, compared to male pharmacists, female pharmacists were less likely to recommend an antibiotic change: 30 (19.0%) of 158 timeouts by female pharmacists versus 52 (38.0%) of 137 timeouts by male pharmacists (*P* < .001). When female pharmacists made recommendations, they were less likely to be accepted by hospitalists than those made by male pharmacists: 10 (33.3%) of 30 versus 41 (78.8%) of 52, respectively (*P* < .001). Thus, female pharmacists were less likely to have an antibiotic timeout result in an antibiotic change: 10 (6.3%) of 158 timeouts by female pharmacists versus 41 (29.9%) of 137 timeouts by male pharmacists (*P* < .001). This difference in recommendations and acceptance by gender was observed for all types of recommendations, with the exception of making a recommendation to stop antibiotics (Fig. 1). Differences by gender, in acceptance of recommendations are shown by diagnosis in Figure 2.

After controlling for patient characteristics, pharmacist gender was significantly associated with whether antibiotic changes were recommended during a timeout (aOR, 0.35; 95% confidence interval [CI], 0.20–0.63) for female versus male pharmacists), whether changes recommended by pharmacists were accepted (aOR, 0.10; 95% CI, 0.03–0.36) for female versus male pharmacists) (see Supplementary Table 1 online for model results), and whether the timeout resulted in an antibiotic change (aOR, 0.15; 95% CI, 0.07–0.33). Neither hospitalist gender nor the interaction between hospitalist and pharmacist gender was significant. When the hospitalist was female, 16 (72.7%) of 22 recommendations were accepted when given by a male pharmacist versus 5 (38.5%) of 13 recommendations given by female pharmacists. Similarly, when the hospitalist was male, 24 (82.8%) of 29 recommendations were accepted when given by a male pharmacist versus 5 (31.3%) of 16 recommendations given by female pharmacists.

Of the 137 timeouts conducted by male pharmacists, our 2 male champions were responsible for 73 of these. The 2 champions were also responsible for 42 (80.8%) of 52 antibiotic recommendations resulting from timeouts. However, the success rate of the male champions (78.6%, 33 of 42) was similar to that of male nonchampions (80%, 8 of 10; *P* > .05). With the champions excluded in a sensitivity analysis, pharmacist gender remained associated with hospitalist acceptance of pharmacist recommendations (aOR, 0.04; 95% CI, 0–0.53; *P* = .02).

## Discussion

In this retrospective analysis of the ROAD Home intervention, pharmacist gender was significantly associated with whether antibiotic changes were recommended during a timeout and whether recommended changes were accepted. Thus, antibiotic timeouts conducted by female pharmacists were substantially less likely (aOR, 0.15) to result in an antibiotic change compared to timeouts conducted by male pharmacists. This discrepancy in intervention effectiveness could have profound effects on whether antibiotic stewardship interventions reach patients and improve their outcomes.

Only 1 in 3 antibiotic recommendations made by female pharmacists were accepted, far fewer than recommendations made by male counterparts (78.8%). This finding held true even after our 2 male champions were removed. Subsequently, pharmacist gender was associated with whether an antibiotic was prescribed at discharge. The potential causes of this gender difference are numerous. In responding to antibiotic stewardship interventions, physicians generally have a choice about whether they accept the recommendation to change an antibiotic prescription. Asymmetry exists in power, authority, and responsibility between pharmacy and medicine with respect to antibiotic decision making.^7^ Unlike other consultants, antibiotic stewardship interventions are typically unsolicited (and may be unwanted) and, depending on the hospital culture, could be perceived as encroaching on physician autonomy.^7^ Antibiotic stewardship as a field often uses interactive interventions that are bolstered by pre-existing collegial relationships between stewards and prescribers.^6,10^ This combination of uninvited recommendations relying on pre-existing relationships in a setting where power differentials already exist creates a context that could exacerbate the effect of unconscious biases on prescriber willingness to accept a stewardship recommendation, including gender bias.

Interestingly, in our primary analysis, female pharmacists were less likely to recommend an antibiotic change during antibiotic timeouts. Prior qualitative studies have shown that pharmacists are strategic about when and where to intervene, saving their recommendations for areas where they believe they are more likely to have success or high-risk scenarios likely to cause imminent patient harm.^19–21^ It is possible that, for female pharmacists who have experienced repeated rejection of their recommendations in the past, the threshold for intervention may be higher. On the other hand, men may have higher risk tolerance and therefore feel more confident making recommendations. One survey of 553 clinical pharmacists found that even after controlling for stewardship training, female pharmacists felt less competent in antibiotic stewardship.^22^ This could be due to differences in self-efficacy and self-confidence by gender or because female pharmacists have different experiences of success in stewardship interventions. This differential in confidence could lead to pharmacist disengagement from antibiotic stewardship if their recommendations are not routinely accepted.

Both of our pharmacist champions were male, despite having more female than male clinical pharmacists working with the hospitalists. Although this study was somewhat underpowered, our champions were not more likely to have their recommendations accepted than nonchampion males (78.8% versus 80%), but they were far more likely to make recommendations (57.6% [42 of 73] versus 15.6% [10 of 64]) and thus had higher overall success in terms of quantity of accepted recommendations.

In the United States, gender biases have been shown to affect other aspects of medical care. For example, male physicians are more likely to refer to male surgeons.^23^ In that study, female physicians were not more likely to refer to male surgeons. That finding contrasts with our study, in which both male and female hospitalists were more likely to accept recommendations by male versus female pharmacists. The difference could be due to the status differential of pharmacists versus surgeons and because surgeon referrals are voluntary, whereas antibiotic stewardship recommendations are unsolicited.

Research in gender bias related to pharmacists is limited. Much like other specialties, female pharmacists earn less than their male counterparts even after adjusting for work characteristics,^24^ though that wage gap may be decreasing.^25^ However, to our knowledge, there have been no studies on the effect of pharmacist gender on pharmacist-led intervention effectiveness. Pharmacy is now a majority female field: 56% of clinical pharmacists are female, a number that has been growing over time.^26^ Given this growth and the importance of pharmacists in areas beyond stewardship (eg, medication reconciliation, transitions of care, patient counselling), there is a critical need to study and mitigate any gender related biases that may exist.

Our study must be taken in the context of limitations. First, though we found no difference in training or years of experience, we were underpowered to adjust for pharmacist characteristics that could impact comfort and knowledge related to making antibiotic recommendations. Much larger studies would need to be conducted to determine the influence and interaction of these factors with gender. Second, this small, single-institution study of clinical pharmacists may not be generalizable to other institutions. Notably, this institution is a “high performing” hospital with low antibiotic overuse at discharge, high workforce diversity, and a history of collaborative relationships between pharmacy and hospital medicine; thus, the effect of pharmacist gender at other institutions could be even greater. Third, we were unable to assess the effect of race, ethnicity, or intersectionality on intervention effectiveness, and bias could have had compounding effects. Fourth, we looked at a single intervention at discharge; it is unclear how gender would affect other antibiotic stewardship or pharmacist recommendations. Similarly, the ROAD Home Intervention relied on clinical pharmacists, rather than infectious diseases pharmacists who could have different experiences and may be less affected by bias due to higher perceived expertise in antibiotic prescribing. Finally, we did not independently review antibiotic recommendations and changes for accuracy, nor could we confirm the accuracy of timeout documentation; self-reported data by pharmacists may lead to biased reporting of results. Study strengths include the ability to assess intervention effectiveness by pharmacist gender and by pharmacist and hospitalist gender concordance and that all participants were blinded to the study objective to evaluate gender bias.

Our findings have several potential implications. First, to our knowledge, ours is the first study to assess the association of pharmacist gender on the effectiveness of any pharmacist intervention, including antibiotic stewardship. The results need to be confirmed in larger studies across diverse settings which would require prospective data collection—potentially including mixed methods—of stewardship intervention effectiveness by pharmacist. However, if gender bias does play a role in whether physicians accept antibiotic stewardship recommendations, it could affect not just the effectiveness of antibiotic stewardship programs but other pharmacy-led interventions. Because antibiotic stewardship can improve antibiotic use and reduce patient harm,^27^ gender bias in pharmacist recommendations could affect patient outcomes. Healthcare systems could consider adding the potential effects of bias on interprofessional team dynamics during implicit bias training. Second, perceptions of bias or ineffectiveness could affect pharmacist satisfaction and burnout, which is already high among stewardship pharmacists.^28^

In conclusion, antibiotic stewardship recommendations made by female versus male pharmacists were less likely to be accepted by hospitalist physicians. These findings suggest that gender bias may play a role in whether physicians accept pharmacist or stewardship recommendations, which could negatively affect antibiotic use and patient outcomes.

## Supplementary Material

Appendix

## Figures and Tables

**Table 1.** Pharmacist Characteristics and Survey Responses

Variable	Men	Women	*P* Value^a^
**Demographics from survey and institutional database**	(N=8)	(N=12)	
Completed a PGY-1 pharmacy residency, no. (%)	5 (62.5)	8 (66.7)	.848
Board certification in pharmacotherapy, no. (%)	3 (37.5)	4 (33.3)	.525
**Demographics From Survey and Online Professional Profiles** ^b^	(N=6)	(N=8)	
Years since completion of terminal degree/post-graduate training; mean (SD)	4.6 (2.2)	3.5 (2.4)	.404
Completed a PGY-2 pharmacy residency, no. (%)	1 (16.6), non-ID	1 (12.5), oncology	.825
**Survey responses**	(N=5)	(N=4)	
**Sexual orientation, no. (%)**			
Heterosexual	4 (80)	4 (100)	
No answer	1 (20)	0	
**Race, no. (%)**			
White	4 (80)	1 (75)	
Arab	1 (20)	0	
Asian American or Pacific Islander	0	1 (25)	
**Ethnicity, no. (%)**			
Hispanic	0	0	
Not Hispanic	5 (100)	4 (100)	
How comfortable are you recommending changes to antibiotic prescriptions if you think guidelines aren’t being met? 1 = very uncomfortable, 5 = very comfortable	4.6 (0.5)	4.8 (0.5)	
Compared to other aspects of your job as a pharmacist, how interested are you in antibiotic stewardship? mean (SD), 1 = very unimportant, 5 = very important	3.4 (0.5)	3.8	
How important do you think antibiotic stewardship is? mean (SD), 1 = very unimportant, 5 = very important	4.8 (0.4)	5.0 (0)	
Please indicate the degree to which you think gender/sex influences how team members respond to your antibiotic stewardship recommendations, answering “some,” “a lot,” or “extreme”, no. (%)^c^	2 (40)	4 (100)	
What percentage of your FTE (total effort) is spent working with (any) clinical teams (ie, on service)? mean % (SD)	57 (29)	70 (17)	
What percentage of your total professional effort is spent working with hospitalists on the hospital medicine service? mean % (SD)	49 (32)	39 (24)	
**Additional pharmacy training, no. (%)**			
Antibiotic stewardship training during PGY-1 year	5 (100)	4 (100)	
Local antibiotic stewardship training by ID pharmacists	3 (60)	4 (100)	
Board certification in infectious diseases	0	0	
SIDP antibiotic stewardship certificate	0	0	
SHEA stewardship certificate	0	0	
MAD-ID basic stewardship program	0	0	
MAD-ID advanced stewardship program	0	0	
CDC training on antibiotic stewardship	0	0	
Other (specific)	0	0	

Note. PGY, postgraduate year; SD, standard deviation; ID, Infectious Diseases; FTE, full-time equivalents; SIDP, Society of Infectious Diseases Pharmacists; SHEA, Society for Healthcare Epidemiology of America; MAD-ID, Making a Difference in Infectious Diseases; CDC, Centers for Disease Control and Prevention.

a Using *t* test or χ^2^ test, as appropriate. *P* < .05 considered significant.

b 6 pharmacists (4 women, 2 men) did not answer the survey and did not have online profiles available. These pharmacists all had roles in the hospital unrelated to the hospital medicine day services (eg, night, operating room, resident); thus, they only conducted timeouts when covering an open shift. In total, they were responsible for only 13 (4%) of 295 timeouts.

c 2 free text comments were provided: (1) “Male colleagues seem to be listened to more than female colleagues (as a very broad generalization)—obviously there are exceptions both ways” and (2) “One (physician’s assistant) in particular.”

**Table 2.** Patient Characteristics Compared by Pharmacist Gender

Variable	All Timeouts (N=295)	Timeouts Conducted by Female Pharmacists (n=158)	Timeouts Conducted by Male Pharmacists (n=137)	*P* Value^a^
Patient age, mean y (SD)	64.2 (16.4)	62.0 (17.0)	66.8 (15.4)	.01
**Patient sex, no. (%)**				.19
Female	152 (51.5)	87 (55.1)	65 (47.5)	
Male	143 (48.5)	71 (44.9)	72 (52.6)	
**Patient race, no. (%)**				.82
White	251 (85.1)	133 (84.2)	118 (86.1)	
Black	31 (10.5)	17 (10.8)	14 (10.2)	
Other^b^	13 (4.4)	8 (5.1)	5 (3.7)	
Charlson comorbidity index, median (IQR)	5 (2–8)	6 (3–8)	5 (2–8)	.15
qSOFA score at 0–24 h, median (IQR)^c^	1 (0–2)	1 (0–2)	1 (0–2)	.71
Length of hospital stay, median d (IQR)	4.3 (2.9–6.7)	4.0 (2.9–6.1)	4.7 (2.9–7.1)	.27
**Infectious disease treated, no. (%)**				.39
Urinary tract infection	83 (28.1)	48 (30.38)	35 (25.6)	
Pneumonia	84 (28.5)	38 (24.05)	46 (33.6)	
Skin and soft-tissue infection	42 (14.2)	26 (16.5)	16 (11.7)	
Bacteremia, osteomyelitis, other	49 (16.6)	26 (16.5)	23 (16.8)	
*Clostridioides difficile* or intra-abdominal infection	37 (12.5)	20 (12.7)	17 (12.4)	
Infectious diseases consultation during hospitalization, no. (%)	72 (24.4)	40 (25.3)	32 (23.4)	.70
**Antibiotic prescribed on discharge, no. (%)**	256 (86.8)	146 (92.4%)	110 (80.3)	<.01
Amoxicillin/Clavulanic acid	82 (32.0)	42 (28.8)	40 (36.4)	.64
Oral cephalosporin	27 (10.6)	19 (13.0)	8 (7.3)	
Fluoroquinolone	46 (18.0)	24 (16.4)	22 (20)	
Sulfamethoxazole/Trimethoprim	29 (11.3)	19 (13.0)	10 (9.1)	
Clindamycin	9 (3.5)	6 (4.1)	3 (2.7)	
Azithromycin	14 (5.5)	9 (6.2)	5 (4.6)	
Doxycycline	17 (6.6)	9 (6.2)	8 (7.3)	
Other	32 (12.5)	18 (12.3)	14 (12.7)	
Antibiotic duration on discharge (patients who received antibiotics only), median d (IQR)	5 (3–10)	6 (3–10)	5 (3–9)	.19
Antibiotic change recommended, no. (%)	82 (27.8)	30 (19.0)	52 (38.0)	<.001
Antibiotic change accepted, no. (%)	51 (17.3)	10 (6.3)	41 (29.9)	<.001
Antibiotic recommendation accepted (primary outcome), n/N (%)	51/82 (62.2)	10/30 (33.3)	41/52 (78.8)	<.001

Note. SD, standard deviation; IQR, interquartile range; qSOFA, quick sequential organ failure assessment score.

a Differences in patient characteristics between male and female pharmacists were evaluated using Pearson χ^2^ or *t* test, as appropriate. *P* < .05 was considered significant.

b Due to small numbers, Asian, Native Hawaiian, Pacific Islander, and “other” are grouped into the “other” category here (see Appendix online for details).

c qSOFA identifies patients outside of the intensive care unit who have a high predicted risk of sepsis-related mortality.
