# Supplementary material for: Pharmacist gender and physician acceptance of antibiotic stewardship recommendations: An analysis of the reducing overuse of antibiotics at discharge home intervention
Source: Infect Control Hosp Epidemiol. Author manuscript; Available in PMC 2023 Dec 28. (PMC10754057; doi:10.1017/ice.2022.136)
Supplement: Appendix [file NIHMS1950521-supplement-Appendix.docx]

Clinical Pharmacist Gender and Physician Acceptance of Antibiotic Stewardship Recommendations: A Retrospective Analysis of the Reducing Overuse of Antibiotics at Discharge Home Intervention

Valerie M. Vaughn, Daniel L. Giesler, Daraoun Mashrah, Adamo Brancaccio, Katie Sandison, Emily S. Spivak, Julia E. Szymczak, Chaorong Wu, Jennifer K. Horowitz, Linda Bashra, Adam L. Hersh

| **Item** | **Page Number** |
| --- | --- |
| **eFigure 1.** ROAD Home Intervention Pocket Card | 1 |
| STROBE Statement | 2 |
| Definition of sex, race, ethnicity | 5 |
| **eTable 1**. Adjusted Model Results | 6 |

**eFigure 1.** ROAD Home Intervention Pocket Card


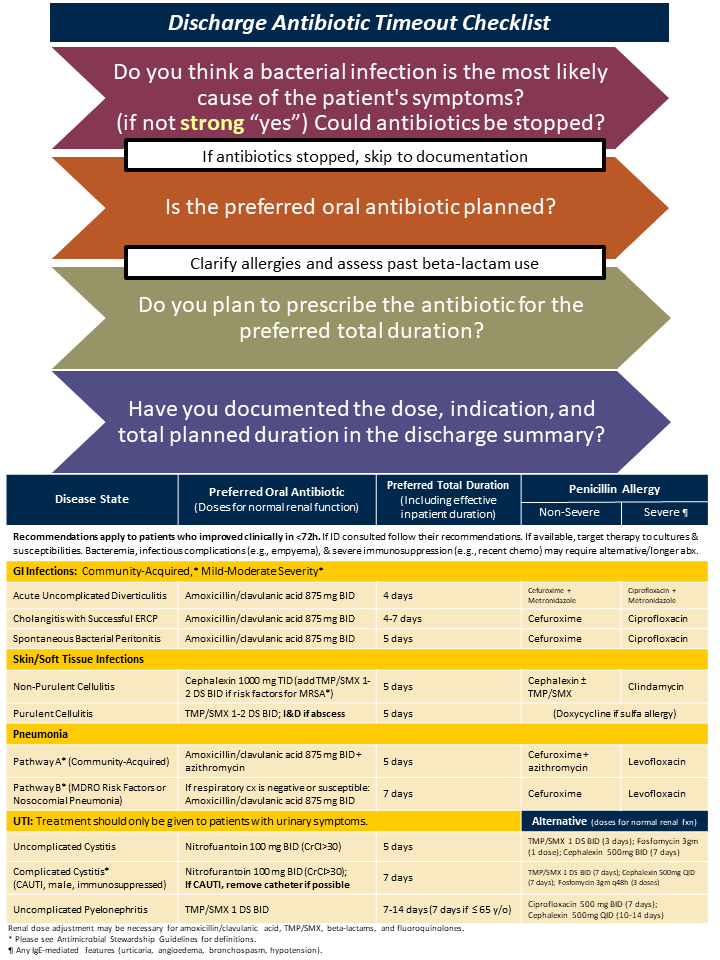


STROBE Statement—checklist of items that should be included in reports of observational studies

|  | Item No | Recommendation | Page  No |
| --- | --- | --- | --- |
| **Title and abstract** | 1 | (*a*) Indicate the study’s design with a commonly used term in the title or the abstract | 1 |
|  |  | (*b*) Provide in the abstract an informative and balanced summary of what was done and what was found | 4 |
| Introduction | | | |
| Background/rationale | 2 | Explain the scientific background and rationale for the investigation being reported | 6 |
| Objectives | 3 | State specific objectives, including any prespecified hypotheses | 6 |
| Methods | | | |
| Study design | 4 | Present key elements of study design early in the paper | 6-7 |
| Setting | 5 | Describe the setting, locations, and relevant dates, including periods of recruitment, exposure, follow-up, and data collection | 6-7 |
| Participants | 6 | (*a*) *Cohort study*—Give the eligibility criteria, and the sources and methods of selection of participants. Describe methods of follow-up  *Case-control study*—Give the eligibility criteria, and the sources and methods of case ascertainment and control selection. Give the rationale for the choice of cases and controls  *Cross-sectional study*—Give the eligibility criteria, and the sources and methods of selection of participants | 7 |
|  |  | (*b*) *Cohort study*—For matched studies, give matching criteria and number of exposed and unexposed  *Case-control study*—For matched studies, give matching criteria and the number of controls per case | n/a |
| Variables | 7 | Clearly define all outcomes, exposures, predictors, potential confounders, and effect modifiers. Give diagnostic criteria, if applicable | 8 |
| Data sources/ measurement | 8* | For each variable of interest, give sources of data and details of methods of assessment (measurement). Describe comparability of assessment methods if there is more than one group | *8* |
| Bias | 9 | Describe any efforts to address potential sources of bias | 9 |
| Study size | 10 | Explain how the study size was arrived at | n/a |
| Quantitative variables | 11 | Explain how quantitative variables were handled in the analyses. If applicable, describe which groupings were chosen and why | n/a |
| Statistical methods | 12 | (*a*) Describe all statistical methods, including those used to control for confounding | 8 |
|  |  | (*b*) Describe any methods used to examine subgroups and interactions | 9 |
|  |  | (*c*) Explain how missing data were addressed | 9 |
|  |  | (*d*) *Cohort study*—If applicable, explain how loss to follow-up was addressed  *Case-control study*—If applicable, explain how matching of cases and controls was addressed  *Cross-sectional study*—If applicable, describe analytical methods taking account of sampling strategy | N/A |
|  |  | (*e*) Describe any sensitivity analyses | 9 |

| Results | | | |
| --- | --- | --- | --- |
| Participants | 13* | (a) Report numbers of individuals at each stage of study—eg numbers potentially eligible, examined for eligibility, confirmed eligible, included in the study, completing follow-up, and analysed | N/A; analysed all patients |
|  |  | (b) Give reasons for non-participation at each stage |  |
|  |  | (c) Consider use of a flow diagram |  |
| Descriptive data | 14* | (a) Give characteristics of study participants (eg demographic, clinical, social) and information on exposures and potential confounders | Table 1 |
|  |  | (b) Indicate number of participants with missing data for each variable of interest | 9 |
|  |  | (c) *Cohort study*—Summarise follow-up time (eg, average and total amount) | n/a; in hospital outcomes |
| Outcome data | 15* | *Cohort study*—Report numbers of outcome events or summary measures over time | *n/a* |
|  |  | *Case-control study—*Report numbers in each exposure category, or summary measures of exposure |  |
|  |  | *Cross-sectional study—*Report numbers of outcome events or summary measures |  |
| Main results | 16 | (*a*) Give unadjusted estimates and, if applicable, confounder-adjusted estimates and their precision (eg, 95% confidence interval). Make clear which confounders were adjusted for and why they were included | 10 |
|  |  | (*b*) Report category boundaries when continuous variables were categorized | n/a; no continuous outcomes |
|  |  | (*c*) If relevant, consider translating estimates of relative risk into absolute risk for a meaningful time period | n/a; OR; also raw outcomes reported |
| Other analyses | 17 | Report other analyses done—eg analyses of subgroups and interactions, and sensitivity analyses | 10-11 |
| Discussion | | | |
| Key results | 18 | Summarise key results with reference to study objectives | 11 |
| Limitations | 19 | Discuss limitations of the study, taking into account sources of potential bias or imprecision. Discuss both direction and magnitude of any potential bias | 13-14 |
| Interpretation | 20 | Give a cautious overall interpretation of results considering objectives, limitations, multiplicity of analyses, results from similar studies, and other relevant evidence | 11-14 |
| Generalisability | 21 | Discuss the generalisability (external validity) of the study results | 14 (needs to be replicated) |
| Other information | | | |
| Funding | 22 | Give the source of funding and the role of the funders for the present study and, if applicable, for the original study on which the present article is based | 17 |

*Give information separately for cases and controls in case-control studies and, if applicable, for exposed and unexposed groups in cohort and cross-sectional studies.

**Note:** An Explanation and Elaboration article discusses each checklist item and gives methodological background and published examples of transparent reporting. The STROBE checklist is best used in conjunction with this article (freely available on the Web sites of PLoS Medicine at http://www.plosmedicine.org/, Annals of Internal Medicine at http://www.annals.org/, and Epidemiology at http://www.epidem.com/). Information on the STROBE Initiative is available at www.strobe-statement.org.

# Definition of sex, race, ethnicity

**PATIENT GENDER**

Instructions: Review the medical record to determine the gender of the patient.

This is a required field and the form cannot be submitted without an entry in this field.

Select one of the following:

- *“Male”* if the patient is categorized as a man in the medical record.
- *“Female”* if the patient is categorized as a woman in the medical record.
- *“Unknown”* if the patient’s gender is unknown.

**RACE**

Instructions: Review the medical record to determine the patient’s race.

Select one of the following:

- *“American Indian or Alaskan Native”* if patient demographic information indicates patient is Native American, American Indian, or Alaska Native.
- *“Arab and Chaldean Ancestries”* if the patient demographic information indicate patient is of Arab or Chaldean Ancestries.
- *“Asian”* if patient demographic information indicates Asian.
- *“Black or African American”* if patient demographic information indicates patient is black or African American.
- *“Native Hawaiian”* if patient demographic information indicates patient is Native Hawaiian.
- *“Pacific Islander”* if patient demographic information indicates patient is Pacific Islander.
- *“White or Caucasian”* if patient demographic information indicates patient is white or Caucasian.
- *“Other”* if patient demographic information indicates the patient is a race other than what is listed above.
- *“Unknown”* if patient’s race is not indicated in the medical record.

| **eTable 1**. Association of Pharmacist Gender with Acceptance of Recommendations, Multivariable Model | | |
| --- | --- | --- |
|  | OR (95% CI) for Acceptance of Pharmacist Recommendation | P-value^a^ |
| Pharmacist Gender, Female | 0.10 (0.03, 0.36) | <.001 |
| Physician Gender, Female | 0.62 (0.19, 2.01) | .427 |
| Patient Age (per additional year) | 1.00 (0.96, 1.04) | .982 |
| Patient Gender, Female | 2.93 (0.88, 9.71) | .084 |
| Patient Race, Non-white^b^ | 1.46 (0.25, 8.61) | .678 |
| Charlson Comorbidity Index (per additional point) | 1.04 (0.87, 1.23) | .683 |
| qSOFA score at 0-24h^c^ (per additional point) | 1 (0, 2) | .761 |
| Infectious disease treated |  |  |
| Skin and soft tissue infection | REF |  |
| Pneumonia | 1.26 (0.21, 7.74) | .804 |
| Urinary Tract Infection | 2.42 (0.3, 19.39) | .409 |
| Bacteremia, Osteomyelitis, Other | 2.17 (0.21, 22.21) | .517 |
| *Clostridioides difficile* or Intraabdominal Infection | 1.53 (0.17, 13.81) | .706 |
| Infectious Diseases Consultation during Hospitalization | 0.53 (0.12, 2.22) | .386 |
| Length of Stay (per additional day) | 1.03 (0.91, 1.17) | .633 |

^a^ To identify the association of pharmacist gender with the success of the antibiotic timeout, we used a logistic regression model controlling for patient characteristics which could impact intervention effectiveness. P<0.05 considered significant.

^b^ Due to small numbers, race was dichotomized as white vs. non-white which includes Black, Asian, Native Hawaiian, Pacific Islander, and “other” (see appendix for details).

^c^ qSOFA identifies patients outside of the intensive care unit who have a high predicted risk of sepsis-related mortality

Abbreviations: SD, standard deviation; IQR, interquartile range; qSOFA, quick sequential organ failure assessment score
